# Professional experiences of spanish advanced practice nurses: qualitative research

**DOI:** 10.1186/s12912-024-02105-6

**Published:** 2024-06-26

**Authors:** Yolanda Cantón-Rodríguez, Olivia Ibáñez-Masero, E. Begoña García-Navarro, Ángela María Ortega-Galán, María Isabel Ventura-Miranda, María Dolores Ruiz-Fernández

**Affiliations:** 1 Department of Nursing, Physiotherapy and Medicine, University of Almería, Almería, Spain; 2 Facultad de Ciencias de la Salud, Universidad Autónoma de Chile, Providencia, Chile; 3 Department of Nursing, University of Huelva, Huelva, Spain

**Keywords:** Advanced practice nursing, Professional competence, Nurse’s role, Nursing care, Qualitative research

## Abstract

**Background:**

Advanced practice nursing has emerged as a result of the evolution of healthcare systems, the changing needs of the population and the academic development of nursing, as well as sociodemographic and epidemiological changes. The aim of this study is to describe the professional experiences of Spanish advanced practice nurses in specific positions within the healthcare system in order to better understand the development and characteristics of this specialised nursing role.

**Methods:**

A descriptive qualitative study was conducted. Fourteen advanced practice nurses from healthcare centres participated. Semi-structured interviews were carried out. Braun and Clarke’s method for reflexive thematic analysis was followed. The Atlas. Ti version 22 program was used for technological support. The COREQ checklist was used to optimise the reporting of this qualitative study.

**Results:**

From the analysis of the data collected, three themes and six subthemes were extracted: 1) Advanced practice nursing on the rise: (a) The driving forces in the development of advanced practice nursing, (b) Barriers to the development of advanced practice nursing; 2) Advanced practice nurses as a response to the population’s needs: (a) The development of a new professional nursing role, (b) The patient at the centre of care in advanced practice nursing; 3) Training as the foundation for advanced practice nursing: (a) Expert nurses in a specific context, (b) Differences in the level of training depending on the context.

**Conclusion:**

Advanced practice nurses have faced countless barriers and difficulties that have impeded them from demonstrating their importance and effectiveness within the healthcare system. A stable regulatory framework for the functions of advanced practice nurses is required to promote care, training and research in the field of advanced practice nursing. Health institutions need to promote the role of advanced practice nurses, facilitate the employment of new professionals, and establish new areas of practice.

**Trial registration:**

Not applicable.

## Introduction

The range of nursing roles is currently diversifying due to the changing needs, demands and expectations of the population. Increasingly specific or advanced nursing positions have emerged for various reasons [1–3]; firstly, as a result of sociodemographic and epidemiological changes (aging, chronicity, comorbidity, progressive increase in the demand for care); secondly, due to the evolution of healthcare systems (technological advances, complexity of the care network, limited resources); and lastly, following the full academic development of undergraduate and postgraduate nursing degrees. One of the strategies to address this scenario is to create advanced practice nurse (APN) positions.

The International Council of Nurses [4, p.9] defines an APN as a “registered nurse who has acquired the expert knowledge base, complex decision-making skills and clinical competencies for expanded practice, the characteristics of which are shaped by the context and/or country in which s/he is credentialed to practice”. APNs develop their competencies in the field of care to respond to the specific needs of the population by reinforcing, extending, or including new services to those already existing in the healthcare system with the aim of improving accessibility, coordination, efficiency, and health outcomes [5]. To this end, they integrate four roles into their practice: clinical expert, providing expert care; consultant, collaborating with the multidisciplinary team and training health professionals; teacher, assuming responsibility for their continuous professional development as well as educational interventions for patients; and researcher, implementing evidence-based practice [4, 5].

## Background

Advanced practices originated in the United States in the mid-20th century with the roles of nurse practitioners and clinical nurse specialists. The US and Canada became countries of reference and the United Kingdom and Europe followed suit [6, 7]. Advanced Practice Nursing is currently expanding across the world. The APN’s role is characterized by its heterogeneity, as the policies and strategies for its implementation differ depending on the country [7]. In Spain, some autonomous communities have included APNs in health care, mainly to deal with chronic diseases: The APN for Urgent Care of Minor Illnesses in Catalonia or the Advanced Clinical Nurse in the Basque Country [7]. In Andalusia, one of the largest communities in Spain, there are APN roles in six areas: diabetology, stomatherapy, oncology, palliative care, chronic wounds, and case management [5]. The latter is the most well-known by the population, as its functions are more established, and it was the first advanced practice nursing role on a regional and national level [7].

Sastre-Fullana et al. conducted an international literature review on the competencies of the APN, including four competency assessment instruments, five APN models and six identified roles [8]. The APNs’ clinical competencies in these roles were assessed and compared [2, 9]. The benefits of their professional activity on patients, the healthcare system and multidisciplinary care have been studied, to a greater or lesser extent, depending on the role, competencies, and training [10], [11], [12]. However, there is a lack of literature on the more personal aspects of this role, including the experiences of APNs in health care. Therefore, the aim of this study is to describe the professional experiences of Spanish advanced practice nurses in specific positions within the healthcare system in order to better understand the development and characteristics of this advanced nursing role.

## Method

### Design and setting of the study

A descriptive qualitative study was conducted. Descriptive qualitative studies faithfully represent the experiences of the participants [13]. They have a low interpretative level and offer readers the possibility to reflect on the reality described so that they can draw their own conclusions. They do not seek the verification of knowledge, but rather the discovery and interpretation of it, based on the participants’ experiences [14, 15]. The COREQ checklist has been used to optimise the reporting of this qualitative study [16].

### Recruitment and characteristics of the participants

The study was conducted in hospitals and primary care centres in the province of Almería between January and April 2023. Purposive and snowball sampling were used to recruit 14 participants, all of whom were APNs working in healthcare centres. The inclusion criteria were to have had a minimum of one year’s experience as an APN and to hold a position in one of the following areas established by the Andalusian Health Service (Spain): diabetology, stomatherapy, chronic wounds, oncology, palliative care, and case management [5]. The exclusion criterion was to not be working as an APN. The subjects were recruited via telephone by the principal investigator, who they did not know, and they were not rewarded in any way. All fourteen subjects agreed to participate. The sample comprised one diabetic nurse, two stomatherapists, two chronic wound care nurses, one oncology nurse, one palliative care nurse, five case managers and two nursing home case managers.

### Description of all processes

#### Data collection

The principal investigator conducted a single semi-structured interview in Spanish with each participant until data saturation was reached because the participants were no longer providing new information [17]. The principal investigator was a female nursing student of the Master’s Degree in Nursing Sciences Research at the University of Almeria, who had been trained by experts in the field. The interviews took place in the healthcare centres where the APNs were working so that the researchers could familiarise themselves with their work environment. The interviews had an average duration of 40–60 min and were recorded with two handheld recorders. A semi-structured interview script was developed with a range of questions drafted by the main researcher and approved by the rest of the team (Table 1). The interview script was practised by the research team beforehand and was piloted during the first interviews. Subsequently, new questions were added to better understand the phenomenon. Based on the emerging design of the qualitative study [17] and the interest of the narrated experiences, the pilot data were included in the main work… After transcription of data, the participants had the opportunity to read the transcripts and add to the information provided by email.Finally, the quotes were translated by a native British professional editor who certified that the intent of the author’s message was not altered in any way.

**Table 1:** Interview script

Interview stage	Objective	Content and sample questions
	Our intentions	I am a student of the Master’s Degree in Nursing Sciences Research and I would like to continue with this line of research for my Final Degree Project on APNs. Understanding your professional experience as an APN would be useful to address the more personal side of the development of advanced aractice nursing.
**Introduction**	Information and ethical considerations	The interview will be audio-recorded for later analysis. Only the research team will have access to these recordings. Participation is voluntary and anonymous. Participants have the right to withdraw from the study at any time.
**Opening**	Consent	Signing of informed consent and verbal consent.
	Opening question	How would you define an advanced practice nurse?
**Continued**	Guided conversation	How do you generally integrate the four main functions of an APN (research, training, practice and management) in your daily work? Do you think you have enough autonomy to carry out your duties? What do you think of the APN-to-patient ratio? Do you think that institutional support for developing APNs is sufficient? Do you think your role is understood and appreciated by the wider population and other healthcare professionals? And by patients?
**Closing**	Final question	What do you think about being one of the first APNs and being part of the growth and development of this role?
	Acknowledgements	Thank you for your participation and for the information provided for the study. Please do not hesitate to contact us if you have any questions. You will receive a copy of the study.

*Source* own elaboration. Data analysis

### Data analysis

Braun & Clarke’s thematic analysis method was followed [18], and the Atlas. Ti version 22 program was used for technological support. The audio of the interviews was recorded with two handheld recorders and transcribed in full, including field notes, following existing transcription standards. Subsequently, a general reading of all the transcripts was conducted (phase 1: familiarization with the data), followed by a more exhaustive reading in which quotes were given codes (phase 2: systematic coding). The codes were grouped with the aim of extracting themes, ensuring that the grouped codes and coded quotes were consistent (phases 3 and 4: generation and development of themes). The most relevant quotes were then selected, and their codes were grouped into six subthemes and three main themes (phase 5: refinement, naming and definition of themes). The data obtained were then analysed, and a data report was created (phase 6: production of the final report). This process was conducted by the principal investigator and reviewed by the other authors.

### Rigor

To ensure rigor, the credibility (triangulation of data), transferability (detailed description of the participants’ perceptions and the context in which the study was conducted), dependability (detailed reporting of methodology) and confirmability (the transcripts and a table summarising the results were given to the participants so that they could corroborate that the findings reflected their experiences) [19]. Furthermore, investigator triangulation was performed: the information collected was analysed separately by the principal investigator and other researchers to identify overlapping themes and reach a unanimous version [19].

### Ethical aspects

The authors respected the Helsinki declaration [20]. The study was approved by the Ethics and Research Committee of the Department of Nursing, Physiotherapy and Medicine of the University of Almeria (code: 232/23). The participants were informed about the aim of the study, the methodology used and their rights. They signed an informed consent form in accordance with the European Personal Data Protection Act that guaranteed confidentiality and anonymity without repercussions in case of withdrawal from the study.

## Results

A total of 14 APNs participated, the majority of whom were women with a mean age of 55 years (SD = 3.77) [Table 2].

**Table 2:** Socio-demographic characteristics of the participants

Participant	Sex	Age	Years of experience as an APN	Additional training
P1	Female	49	2	Advanced university course
P2	Female	55	17	Master’s degree
P3	Female	56	17	Psychology Degree
P4	Male	55	21	2 Bachelor’s Degrees (Psychology and advanced university course)
P5	Female	52	3	Master’s degree
P6	Female	51	6	Community Nursing Specialty
P7	Female	61	10	Master’s degree
P8	Female	52	11	Bachelor’s Degree in Social Work and Community Nursing Specialty
P9	Female	49	9	Master’s degree
P10	Female	58	2	Master’s degree
P11	Male	59	1	Nursing Degree
P12	Male	56	8	Doctoral degree-(Ph.D)
P13	Male	55	3	Master’s degree
P14	Female	59	5	Nursing Degree

*Note* Advanced university course: Higher postgraduate studies whose main objective is to provide professional training highly focused on practical application in the labour market so that the student specializes in a specific area. Postgraduate certified. *Source* own elaboration

From the analysis of the data collected, three main themes were extracted, which were divided into two subthemes with the aim of describing the APNs’ professional experiences (Table 3).

**Table 3:** Table of themes, sub-themes and codes extracted from the analysis

THEME	SUBTHEME	CODES
Advanced practice nursing on the rise	The driving forces in the development of advanced practice nursing	Rewarding, work, enthusiasm, motivation, passion, vocation.
	Barriers to the development of advanced practice nursing	Opening up the field, lack of legal framework, difficult start, demonstrating importance, mistrust, lack of knowledge, difficult, difficulty to change, uncertainty, struggle, fear, rejection of nursing.
Advanced practice nurses as a response to the population’s needs	The development of a new professional nursing role	Novelty, new competencies, responsibility, piloting, project, all-round nurses, previous experience, expertise.
	The patient at the centre of care in advanced practice nursing	Quality improvement, social need, patient-centred care, complex patients, holistic care.
Training as the foundation for advanced practice nursing	Expert nurses in a specific context	Specific field, knowledge, training, further training, advanced practice-based training, specific training, integrating knowledge into practice.
	Differences in the level of training depending on the context	Self-taught, lack of training support, prior initial training, institutional training, sufficient training, financial investment in training.

*Source* Own elaboration

## Advanced practice nursing on the rise

Advanced practice nursing is characterized by the obstacles that APNs have had to face. These nurses have responded to the emerging and changing needs of a society that has limited resources yet demands high-quality service. There is little awareness of the APN’s role, which is even dismissed by part of the nursing community. Due to the absence of a legal framework to support them, APNs have been forced to demonstrate the importance of their role time and time again, as well as open the field to new APNs. Together with the difficulty of integrating changes into the healthcare system, this has resulted in the APNs facing obstacles from the very beginning. Indeed, the initial phase of their careers was characterized by mistrust, uncertainty and fear. Despite this, the APNs were driven to develop the field thanks to their vocation, motivation, enthusiasm, and gratitude. Two opposite but complementary sub-themes emerge from this theme: the *driving forces* and the *main barriers to the development* of advanced practice nursing:

### Subtheme 1: the driving forces in the development of advanced practice nursing

The APNs described how their vocation was a driving force when they accepted the role. They faced difficulties with enthusiasm and motivation, staying focused on how their work would improve their patients’ quality of life and care. Professional satisfaction has been acknowledged as a driving force for developing advanced practice nursing.

*“It’s very exciting. You can actually end up with a passion for wound care. To be able to keep progressing, all the work I did, the extremely specific roles that I undertook, all related to patients with complex chronic wounds, it was extremely rewarding”.* (P12)

*“I was very motivated when I started out with patients who needed me because they are highly complex…I had to learn a lot, but it was all very positive. I remember it very well, a very nice stage in my career.”* (P6).

### Subtheme 2: barriers to the development of advanced practice nursing

The APNs interviewed recounted the difficulty of starting in the position as they encountered systematic barriers that impeded them from doing their job properly. They felt that they had to demonstrate the importance of their role constantly in order to gain a foothold in the community, all while struggling with the great difficulties of implementing and maintaining changes in the healthcare system over time.

*“We are being asked to work efficiently, and to a high standard, and it feels like we have to be demonstrating that our work is important, that it is effective, and for that to happen we need to do it to a high standard.”* (P8).

In addition, some participants described how APNs were rejected by the nursing community, who did not see the need for them despite objective results demonstrating that their work increased quality of care and patient satisfaction. This was exacerbated by a lack of awareness surrounding their functions and the absence of a legal framework to define them.

*“At the beginning, we were somewhat rejected by our nursing community colleagues because it was not very clear what our function was; it was a bit ambiguous. Our colleagues thought that rather than having a case manager, they should add another nurse to the team so that we all work less or that the work is more evenly distributed. Although there was a genuine need for us, it had not been made visible and at the beginning, it was rejected”.* (P3)

## Advanced practice nurses in response to the population’s needs

The APN position emerged in response to the needs of the population. This new professional role was piloted with versatile nurses who had previous experience and expertise. They acquired new competencies and responsibilities to meet society’s need for a role focused on the holistic care of complex patients. This enabled them to place the patient at the centre of care and improve the quality of care provided. There are two sub-themes associated with this topic:

### Subtheme 1: the development of a new professional nursing profile

The APNs had one notable characteristic in common when they were offered the position, which were the years of experience and background in the specialty. The nurses explained how this led them to acquire unique competencies that would allow them to develop the role of the APN.

“*Well, I worked in surgery, as I told you, I started 26 years ago… There were patients undergoing ostomy surgery, of course, long before that and much earlier. The thing is that the patient went home, the surgeon prescribed the device, but there was no follow-up or control of the patient afterwards*.” (P9).

### Subtheme 2: the patient at the Centre of Care in Advanced Practice nursing

Advanced practice nurses attend to patients with complex chronic diseases who require more specialized care and ongoing monitoring. They receive holistic care, in which their biopsychosocial needs take precedence over their conditions.

“*And because it was necessary. It was necessary because we had a number of people with very complex conditions with families who are overwhelmed. The system is swamped, and we needed someone to take these people by the hand and lead them through the healthcare system*.” (P11).

## Training as the foundation for advanced practice nursing

APNs must be experts in a given context. Therefore, one of the fundamental pillars for the development of advanced practice nursing is continuous training, which is needed for acquiring and integrating knowledge into practice in such specific fields. The level of training differs depending on the context. While some APNs received specific, advanced, practice-based training before performing their professional duties, others perceived a total lack of support from institutions in providing useful training resources. This resulted in them having to teach themselves, which can require making a large financial investment. Moreover, they had to start out in their new position without any previous training and had to learn on the job, meaning that they were not able to fulfil their duties properly in the first few years in the role. Two complementary sub-themes have been drawn from the theme of training as a foundation for working in advanced practice nursing:

### Subtheme 1: Expert nurses in a specific context

The APNs described how they had to train extensively to reach the level of expertise required to do their specific job properly. Despite all the years of experience they had prior to advanced practice nursing, the APNs had to reach a minimum level of accredited training to be able to put all the competencies that define them in clinical practice to use.

“*The APN is a nurse who has the knowledge and training in a specific context of advanced practice and who is able to integrate all that knowledge into his/her work.”* (P2).

“*A nurse who has received postgraduate training to become an expert in a subject works independently and gives the patient autonomy. In other words, you are an expert in a specific subject, and you have training*.” (P7).

### Subtheme 2: differences in the level of training according to context

The training that APNs receive when they decide to accept the job varies according to the nursing specialty. Some APNs received prior training that they deemed sufficient and adequate to be able to conduct their new functions.

“*The training process we have undergone has been very good because since we joined last year up until now, we have had continuous professional development… our organization has been training us on various different topics in relation to case management, so I think our training has been quite good”.* (P11)

However, other APNs explained how they felt that their training was insufficient to deal with such complex patients, especially considering that they were being trained when already in the job. As they were aware of their need for training to do their job properly, they taught themselves, despite feeling that it was not right to have to invest their own money when their institution should be providing them with sufficient training.

*“There are very vigorous training programs, but you have to teach yourself. You have to be involved in your clinical practice; you have to be involved in your training”.* (P6)

*“I have not been to a case management course for a long time. Unless you want to do the course through the Andalusian School of Public Health, which costs a lot of money. I am not going to spend that kind of money to do it*”. (P8)

**Fig. 1:**
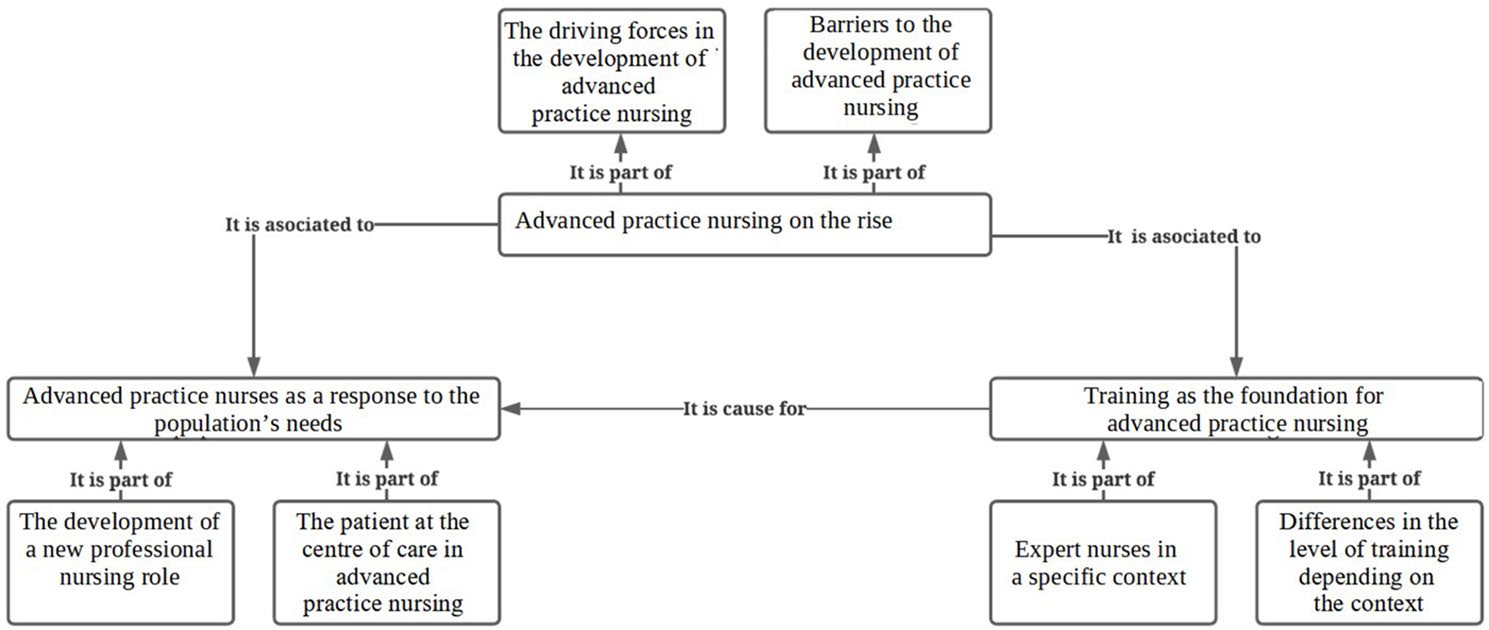
Table of themes and subthemes. (Source own elaboration)

The rise of advanced practice in recent years has been characterized by driving forces and obstacles. These nurses have tried to respond to the needs of the population through the development of a new professional role that places the patient as the centre of care and that relies on expert training in a specific context (Fig. 1).

## Discussion

This descriptive qualitative study aimed to describe the experiences of APNs regarding the development of this professional role within the health system. Advanced practice nursing is developing in response to the changing needs of the population. Hansen & Dysvik explain how these professionals cater for the needs of complex patients by providing services in a given context, thus making the healthcare system more sustainable [21]. With regard to the driving forces in the development of advanced practice nursing, Gysin et al. [22] explain how nurses in Switzerland considered themselves pioneers in shaping the role of advanced practice nurses. They required a visionary mindset to lead them through a process of trial and error that would allow them to guide future generations of nurses and develop the healthcare system [22]. Andalusia, an autonomous community in Spain, has been a pioneer in promoting advanced practice nursing [5]. The pilot projects (diabetology, stomatherapy, oncology, palliative care, chronic wounds, and case management) that have been developed there over the last twenty years have become a model for the rest of Spain [5]. Due to the obstacles faced by APNs, advanced practice nursing has been developed in a heterogeneous way worldwide [8]. Given the pioneering nature of their role and the lack of legal framework to define it, APNs have had to deal with a high level of responsibility and pressure [22]. Nigenda et al. state that the biggest barrier to the development of APNs in Mexico is the country’s own legislation; for advanced practice nursing to be established, there needs to be a policy that first ensures that nurses are safe and supported to be able to carry out their advanced functions autonomously [23].

On the other hand, this new professional nursing role places the patient at the centre of care, resulting in numerous benefits in terms of care: APNs have more time to provide one-to-one care to their patients [22]; patients often find themselves less inhibited to address certain issues [22, 24]; patients’ quality of life improves in terms of physical comfort [25]; increased safety of care [25] and patient satisfaction [26]; increased adherence to medical treatments [26]; and facilitated access to healthcare services [24].

For these benefits to materialise, APNs require expertise in a specific context, thus making training the foundation of advanced practice nursing [8]. APNs require continuous professional development to maintain their competencies and provide safe patient care [27, 28]. There is no consensus on the type of training needed for these nurses. However, in the vast majority of countries in which the role exists, a master’s degree is the minimum requirement [26], and even higher levels of postgraduate education are recommended to enhance clinical skills [22, 29]. This is sometimes a barrier for nurses, as accessing such specific levels of training is not easy, and they encounter inequalities in the process [23, 30]; the results of the study show that more than half of the participants lack the level of mastery required. In addition, APNs are still not remunerated for the roles and competencies they are required to fulfil and sometimes need to pay for their own training. For example, Devictor et al. state that in France, nurses do not receive financial compensation for activities such as case management, training, or research, thus limiting their involvement in the field of advanced practice nursing [31].

This study has some limitations. First, a limited number of APNs in a single cultural and professional context participated in the study. This was due to the scarcity of specific positions for APNs and the lack of an established legal framework to define them. On the other hand, the participants cover a range of APN roles, which enriches the study. Second, the participants were mostly women, which could have led to gender bias in relation to their perceptions. However, given that most nurses are women, this reflects the reality of the profession. Finally, the interviews were conducted when the nurses were busy at work, which may have influenced their accounts. Nevertheless, the participants spent as much time as needed with the interviewer, and their statements were negative in objectively demonstrable aspects. For future lines of research, regulatory policies should be addressed more comprehensively to create a stable legal framework, support training, and enhance research. Furthermore, a larger and more diverse sample should be recruited to improve the generalizability of the results.

## Conclusion

The establishment and development of advanced practice nursing has been characterized by its heterogeneity and barriers such as: the lack of a legal framework to define professional roles and competencies, a lack of financial recognition and mistrust from other nursing colleagues, and the need for specific training and postgraduate education, among others. These nurses have faced a constant struggle to demonstrate their importance and effectiveness within the health system. However, although there is much work to be done, the APNs have been able to carve out a niche for themselves within a system that needs expert nurses to deal with patients with complex chronic diseases in a comprehensive and holistic way, leading to improved quality of care and a reduced burden on an overstretched healthcare system.

The expertise of APNs has led to progress in the health care of patients with complex chronic diseases. These nurses objectively demonstrated that their work has benefits on patient quality of care and satisfaction. Therefore, there is a need to conduct further research to define, develop and promote the role of advanced practice nurses. Furthemore, a stable regulatory framework establishing the functions of advanced practice nurses is required to promote care, training and research in the field of advanced practice nursing. The support of public administrations and the creation of new positions for these nurses in different areas would contribute to a more specialised nursing workforce and care that is more in line with the health needs of today’s population.

## Data Availability

Data are available from the first author or corresponding author on reasonable request.

## References

[1] Ramírez P, Hernández Ó, De Ormijana AS, Reguera AI, Meneses M (2002). Enfermería De práctica avanzada: historia y definición. Enferm Clin.

[2] Rodríguez-Calero MÁ, Villafáfila-Gomila CJ, Sastre-Fullana P (2019). Advanced practice nurses and evidence-based practice. An opportunity for change. Enferm Clin (English Edition).

[3] Robles NL. Estrategia de Cuidados de Andalucía: nuevos retos en el cuidado de la ciudadanía: Resumen ejecutivo.Cons Salud Serv Andaluz Salud[Internet].2015. https://www.sspa.juntadeandalucia.es/servicioandaluzdesalud/sites/default/files/sincfiles/wsas-media-mediafile_sasdocumento/2019/resumen_ejecutivo.pdf. Accessed 18 May 2023.

[4] International Council of Nurses. Guidelines on advanced practice nursing. Geneva: ICN. 2020. https://www.icn.ch/system/files/documents/2020-04/ICN_APN%20Report_EN_WEB.pdf. Accessed 20 Sept 2023.

[5] Lafuente-Robles N, Fernández-Salazar S, Rodríguez-Gómez S, Casado-Mora MI, Morales-Asencio JM, Ramos-Morcillo AJ (2019). Competential development of nurses in the public healthcare system of Andalucía. Enferm Clin (English Edition).

[6] Schober M. Development of advanced practice nursing: the international context. Enferm clin (English Edition). 2019;2019. 10.1016/j.enfcli.2018.08.002. 10.1016/j.enfcli.2018.08.002 30201468

[7] San Martín-Rodríguez L, Soto-Ruiz N, Escalada-Hernández P (2019). Academic training for advanced practice nurses: International perspective. Enferm Clin (English Edition).

[8] Sastre-Fullana P, De Pedro-Gómez JE, Bennasar-Veny M, Serrano-Gallardo P, Morales-Asencio JM (2014). Competency frameworks for advanced practice nursing: a literature review. Int Nurs Rev.

[9] Hako L, Turunen H, Jokiniemi K (2023). Advanced practice nurse capabilities: a mixed methods systematic review. Scand J Caring Sci.

[10] Gutiérrez-Rodríguez L, García-Mayor S, Cuesta-Lozano D, Burgos-Fuentes E, Rodríguez-Gómez S, Sastre-Fullana P (2019). Competencias en enfermeras especialistas y en Enfermeras De Práctica Avanzada. Enferm Clin (English Edition).

[11] Eriksson I, Lindblad M, Möller U, Gillsjö C. Holistic health care: patients’ experiences of health care provided by an Advanced Practice nurse. Int J Nurs Pract. 2018. 10.1111/IJN.12603. 10.1111/ijn.12603 PMC5813192 29071766

[12] Sánchez-Gómez MB, Ramos-Santana S, Gómez-Salgado J, Sánchez-Nicolás F, Moreno-Garriga C, Duarte-Clíments G (2019). Benefits of Advanced Practice nursing for its expansion in the Spanish context. Int J Environ Health Res.

[13] Kim H, Sefcik JS, Bradway C (2017). Characteristics of qualitative descriptive studies: a systematic review. Res Nurs Health.

[14] Toro AG. Lectura crítica De Un Estudio Cualitativo Descriptivo. Index De Enfermería. 2003;40(41):51–7. http://www.index-f.com/index-enfermeria/40-41revista/40-41_articulo_51-57.php. Accessed 18 May 2023.

[15] Sandelowski M (2010). What’s in a name? Qualitative description revisited. Res Nurs Health.

[16] Tong A, Sainsbury P, Craig J (2007). Consolidated criteria for reporting qualitative research (COREQ): a 32-item checklist for interviews and focus groups. Int J Qual Health Care.

[17] Glaser BG, Strauss AL. No title. The Discovery of grounded theory. Strategies for Qualitative Research.,[e-book] Aldine de Gruyter; 1967.

[18] Braun V, Clarke V (2006). Using thematic analysis in psychology. Qual Res Psychol.

[19] Lincoln Y, Guba E (1985). Naturalistic inquiry.

[20] World Medical Association (2013). World Medical Association Declaration of Helsinki: ethical principles for medical research involving human subjects: ethical principles for medical research involving human subjects. JAMA.

[21] Hansen BS, Dysvik E (2022). Expanding the theoretical understanding in Advanced Practice nursing: framing the future. Nurs Forum.

[22] Gysin S, Sottas B, Odermatt M, Essig S (2019). Advanced practice nurses’ and general practitioners’ first experiences with introducing the advanced practice nurse role to Swiss primary care: a qualitative study. BMC Fam Pract.

[23] Nigenda G, Lee G, Aristizabal P, Walters G, Zárate-Grajales RA (2021). Progress and challenges for advanced practice nursing in Mexico and the United Kingdom. J Nurs Manag.

[24] Woo B, Koh K, Zhou W, Wei T, Lopez V, Tam W (2020). Understanding the role of an advanced practice nurse through the perspectives of patients with cardiovascular disease: a qualitative study. J Clin Nurs.

[25] Grešš-Halász B, Dimunová L, Rónayová I, Knap V, Lizáková Ľ (2021). Advanced practice nursing in cardiology: the Slovak perspective for the role development and implementation. Int J Environ Health Res.

[26] Xu C, Koh KWL, Zhou W (2022). The development of advanced practice nurses in Singapore. Int Nurs Rev.

[27] Josi R, Bianchi M (2019). Advanced practice nurses, registered nurses and medical practice assistants in new care models in Swiss primary care: a focused ethnography of their professional roles. BMJ Open.

[28] Chair S, Wong F, Bryant-Lukosius D (2023). Construct validity of advanced practice nurse core competence scale: an exploratory factor analysis. BMC Nurs.

[29] Hanks RG, Eloi H, Stafford L (2019). Understanding how advanced practice registered nurses function as patient advocates. Nurs Forum.

[30] Chau J, Lo S, Lam S (2022). Critical elements in nursing graduates’ transition to advanced practice roles and their perceived impact on patient care: an exploratory, descriptive study of graduates’ and their managers’ perceptions. BMC Nurs.

[31] Devictor J, Burnet E, Henriot T, Leclercq A, Ganne-Carrie N, Kilpatrick K (2023). Implementing advanced practice nursing in France: a country-wide survey 2 years after its introduction. Nurs Open.

